# Supplemental vibrational force does not reduce pain experience during initial alignment with fixed orthodontic appliances: a multicenter randomized clinical trial

**DOI:** 10.1038/srep17224

**Published:** 2015-11-27

**Authors:** Neil R. Woodhouse, Andrew T. DiBiase, Spyridon N. Papageorgiou, Nicola Johnson, Carmel Slipper, James Grant, Maryam Alsaleh, Martyn T. Cobourne

**Affiliations:** 1 King’s College London Dental Institute, Department of Orthodontics, London SE19RT, UK; 2 Brighton and Sussex University Hospitals NHS Foundation Trust, Royal Alexandra Children’s Hospital, Department of Orthodontics, Brighton BN25BE, UK; 3 East Kent Hospitals University NHS Foundation Trust, William Harvey Hospital, Department of Orthodontics, Ashford TN240LZ, UK; 4 University of Bonn, Department of Orthodontics and Department of Oral Technology, Bonn DE-53111, Germany

## Abstract

This prospective randomized trial investigated the effect of supplemental vibrational force on orthodontic pain during alignment with fixed-appliances. Eighty-one subjects < 20 years-old undergoing extraction-based fixed-appliance treatment were randomly allocated to supplementary (20-minutes/day) use of an intra-oral vibrational device (AcceleDent^®^) (n = 29); an identical non-functional (sham) device (n = 25) or fixed-appliances only (n = 27). Each subject recorded pain intensity (using a 100-mm visual-analogue scale) and intake of oral analgesia in a questionnaire, following appliance-placement (T1) and first-adjustment (T2) for 1-week (immediately-after, 4, 24, 72-hours and at 1-week). Mean maximum-pain for the total sample was 72.96 mm [SD 21.59; 95%CI 68.19–77.74 mm] with no significant differences among groups (P = 0.282). Subjects taking analgesics reported slightly higher maximum-pain although this was not significant (P = 0.170). The effect of intervention was independent of analgesia (P = 0.883). At T1 and T2, a statistically and clinically significant increase in mean pain was seen at 4 and 24-hours, declining at 72-hours and becoming insignificant at 1-week. For mean alignment-rate, pain-intensity and use of analgesics, no significant differences existed between groups (P > 0.003). The only significant predictor for mean pain was time. Use of an AcceleDent vibrational device had no significant effect on orthodontic pain or analgesia consumption during initial alignment with fixed appliances.

Pain is a common consequence of orthodontic treatment with fixed appliances and this is often most significant immediately following appliance placement^1^,^2^,^3^. Studies have shown that pain generally increases during the first 24 hours after the appliance has been fitted and then gradually reduces over the following week^3^,^4^,^5^,^6^,^7^,^8^,^9^. This cycle is often repeated as the teeth align and progressively stiffer archwires are placed, which can affect routine day-to-day activities, such as eating and sleeping; and often requires the consumption of oral analgesia^3^,^10^. Indeed, there is evidence that orthodontic pain has the potential to negatively impact on some aspects of treatment, including compliance^11^, likelihood of completion^12^,^13^ and overall outcome^14^.

Although conventional oral analgesics are effective in reducing orthodontic pain^15^, medications can be associated with side-effects and are contraindicated in some individuals. Given these potential negative factors, a number of studies have evaluated different interventions and their relationship to orthodontic pain—particularly variation in appliance design. There has been some focus on pain associated with self-ligating versus conventional brackets during initial alignment, but little evidence exists of clinically significant differences between these designs^7^,^8^,^9^,^16^,^17^, although the process of archwire insertion and removal can be more painful when using 3M Smartclip^TM^ self-ligating brackets^16^,^17^. In terms of aligning archwires, martensitic-active copper nickel-titanium has been associated with greater pain intensity compared with martensitic-stabilized^18^,^19^ and superelastic nickel-titanium has a significantly higher peak pain when compared to multistrand stainless steel^20^; however, the effects of nickel-titanium type appears to be marginal in other studies^4^,^20^ and overall, more evidence is needed^21^,^22^. Low-level laser therapy has also been described as a non-thermal biostimulatory pain-relieving adjunct to fixed appliance treatment^23^,^24^,^25^,^26^,^27^,^28^,^29^ and whilst showing some promise, current evidence remains weak^30^,^31^.

There is evidence that vibrational stimulation can be effective in providing relief for various types of acute and chronic musculoskeletal pain^32^,^33^, sinus pain^34^ and indeed, pain of dental origin^35^. The pain-relieving effects of vibratory stimulation may be achieved by increasing vascularity and reducing areas of ischemia and through the activation of large-diameter sensory nerve fibers^36^. The concept of vibratory stimulation as a method of reducing pain following the adjustment of orthodontic appliances has also been described^37^. A number of devices are now commercially available that have been designed to provide cyclic vibratory force directly to the dentition as an adjunct to orthodontic treatment. Amongst these, AcceleDent® is a prescription-only removable, hands-free device that provides a vibrational frequency of 30 Hz and force of 0.2 N to the dentition (Fig. 1). The patient gently bites onto a vibrating thermoplastic wafer for around twenty-minutes per day as a supplement to their orthodontic treatment. Although these appliances are marketed as a viable method of reducing pain during orthodontic treatment, there is currently little evidence to support this^38^.

The aim of this randomized clinical trial was to investigate the effect of supplemental vibrational force with the AcceleDent appliance on pain during early tooth alignment with fixed orthodontic appliances. The null hypothesis is that supplemental vibrational force does not reduce pain associated with this phase of fixed appliance treatment.

## Methods

### Trial design and changes after trial commencement

This investigation reports a three-arm parallel-randomized controlled trial comparing the effects of supplemental vibrational force on orthodontic pain during initial alignment with fixed appliances. Ethical approval was obtained from the United Kingdom National Research Ethics Service (South East London REC 3: 11/LO/0056) and written informed consent was received from all parents, guardians and children. All methods were performed in accordance with the approved guidelines and regulations. The trial was registered at the European Clinical Trials Database (EudraCT) (2014-004211-37) on 29 September 2014 and ClinicalTrials.gov (NCT02314975) on 25 November 2014. No changes to the methodology occurred following trial commencement. We report and present data according to the CONSORT statement^39^.

### Participants, eligibility criteria, and settings

Participants were recruited from King’s College London Dental Institute (Guy’s Hospital) UK; Royal Alexander Children’s Hospital, Brighton, UK; and William Harvey Hospital, Ashford, UK between July 2011 and May 2014. Eligibility included the following criteria: (1) under 20 years of age at treatment start; (2) no medical contraindications, including regular medication; (3) in the permanent dentition; (4) mandibular arch incisor irregularity; (5) extraction of mandibular first premolars as part of the orthodontic treatment plan.

### Interventions

Participants were randomly assigned to one of three treatment groups: (1) Pre-adjusted edgewise fixed-appliance treatment with adjunctive daily use of a functional AcceleDent® (OrthoAccel® Technologies, Inc, Houston, Texas, USA) vibrational device (Accel-group); (2) Pre-adjusted edgewise fixed-appliance treatment with adjunctive use of a non-functional (sham) AcceleDent device (Accel-sham); and (3) Pre-adjusted edgewise fixed-appliance treatment alone (Fixed-only group). Subjects allocated to functional or sham devices were given direct verbal and written instruction on operation and usage, told to use the device for 20 minutes per day and shown that an electronic timer was part of the device, therefore allowing compliance monitoring. The sham device was identical to the active device in all respects, except that it did not vibrate. The bonding method and fixed appliance was standardized between groups (MBT prescription pre-coated 3M Victory series, 3M Unitek, Monrovia, USA). After bracket bonding, a pre-determined sequence of 0.014-inch and 0.018-inch nickel titanium archwires was used during the period of study. Archwires were inserted and ligated from first molar to first molar using conventional elastomerics. Archwire progression occurred only if full bracket engagement was achievable, which required the relevant archwire to be fully tied into the base of the bracket slot adjacent to each tie wing using elastomeric ligation. All archwires were cut distal to the first molars and were not cinched. No bite planes, auxiliary arches, inter-maxillary elastics, headgears or temporary anchorage devices were used during the period of investigation. All subjects were treated by consultant orthodontists (ATD, NJ, CS, JG); or specialist registrars (NRW, MA) under their direct supervision.

### Primary and secondary outcomes

Subjects were given a pain questionnaire to complete over the week following placement of the fixed appliance and insertion of the initial 0.014-inch nickel-titanium archwire (T1); and following insertion of a 0.018-inch nickel-titanium archwire (T2). The questionnaire recorded orthodontic pain immediately after, 4 hours, 24 hours, 3 days and 1 week following the appointment by means of a 100-mm visual analogue scale (VAS) using the terms “very comfortable” and “very uncomfortable” as peripheral weightings^40^. The VAS score is the distance from the left end of the line to the point of the subjects mark, measured to the nearest millimeter. Each VAS score was measured on two separate occasions by the same operator (NRW); with the mean taken as the representative value. In addition to the VAS score, subjects noted the consumption of oral analgesia during the period of observation. Each subject was free to take non-prescription oral analgesia, as required. The pain questionnaire was completed by the subject and returned at the following appointment. Tooth alignment was calculated using an irregularity-index, which measured the horizontal linear contact-point displacement of each mandibular incisor from the adjacent tooth and therefore represented the sum of the five individual displacements^41^. Rate of initial alignment was calculated as the difference in irregularity-index of casts taken at T1 and T2 divided by the number of days between the two measurements and has been reported elsewhere^42^.

The primary outcome measure for this part of the trial was maximum pain experience during initial alignment with fixed orthodontic appliances, whilst secondary outcomes were (1) mean pain; (2) alignment rate; (3) use of oral analgesia; and (4) number and type of oral analgesia taken during this period of treatment. There were no changes to outcomes following trial commencement.

To examine measurement reliability, 30 sets of VAS scores were selected and re-measured blind two weeks following the original measurement by one assessor (NRW) and the intra-class correlation coefficient was calculated. Repeatability of the measurements was found to be almost perfect (coefficient and its 95% CI included only values above 0.99).

### Sample size calculation

Sample size calculation for this trial was based upon the outcome of initial rate of orthodontic tooth alignment, which gave a required sample of 23 per group and has been described previously^42^. A previous investigation of orthodontic pain using fixed appliances in two groups of UK subjects during initial alignment adopted a difference of 20 mm on the VAS scale as being clinically significant^7^. *Post hoc* power analysis, using a three-group one-way ANOVA to identify the above-mentioned difference, with a root mean square error of 21.51 mm (originating from results of the present trial) and a significance of 0.05, indicated that this trial would yield a power between 82.9% and 89.6%.

### Interim analyses and stopping guidelines

Not applicable.

### Randomization

The randomization sequence was generated by one investigator (MTC) using GraphPad online software (http://www.graphpad.com/quickcalcs/index.cfm) with unrestricted equal participant allocation (1:1:1) undertaken centrally at King’s College London, independently from the clinical operators, following recruitment (allocation concealment)^43^.

### Blinding

Treating clinicians and subjects could not be blinded to the use of AcceleDent; however, subjects were blinded to the allocation of functional or sham appliances, as both were identical in appearance (with the exception that the sham appliance did not vibrate). The pain questionnaires and extracted data were coded appropriately, so that both outcome assessor (NRW) and statistician (SNP) were blinded to subject allocation. The coding of the data was broken after the end of the analysis and no breach of blinding was identified.

### Statistical analysis

We report and present data according to the relevant CONSORT statement^39^. Demographic and clinical characteristics were investigated with conventional descriptive statistics. Differences between groups for single time-points were initially identified among groups with independent t-tests, Analyses of Variance (ANOVAs) and chi-square tests after checking for homoscedacity and normality of residuals. The effect of the intervention on primary and secondary outcomes was investigated using multivariable linear or logistic regression modeling with robust standard errors, adjusted for any possible confounders, including demographics (sex, age) and clinical characteristics (mandibular incisor irregularity at T1). Analyses were also adjusted for the use of oral analgesia and alignment rate between T1-T2, whenever possible. In the analysis of mean pain across time-points, the model accounted for within-patient or time-point correlations. Residuals analysis was conducted to confirm no violation of the linear regression assumptions. All analyses were carried out using Stata 12.0 (Statacorp, College Station, TX, USA). A 2-tailed P-value of 0.05 was considered statistically significant with a 95% confidence interval (CI) for all tests. Crude Bonferroni adjustments of the significance level were conducted for the analyses of the secondary outcomes, which were regarded as exploratory^44^.

## Results

### Participant flow

**Figure 2:**
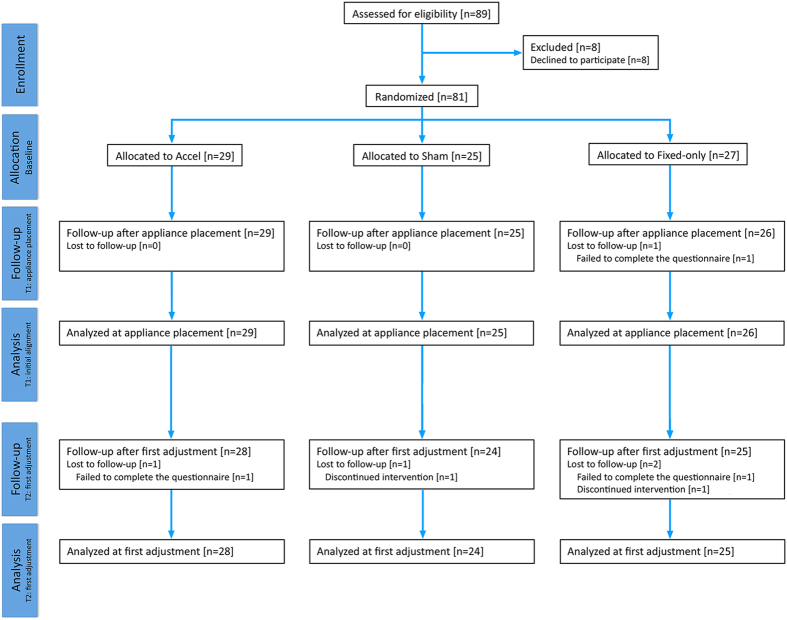
CONSORT diagram showing the flow of subjects through the trial. Note that data were lost at T1 for 1 subject (^#^11 allocated to fixed-only, but this person remained in the trial and T2 data was obtained). Note that subjects ^#^22 allocated to Accel and ^#^26 allocated to Accel-sham also discontinued the intervention but after the collection of T2 pain data. This has been previously reported as final alignment data was not obtained for these individuals^42^.

This three-arm parallel-randomized controlled trial included 81 participants (40 males and 41 females) with a mean age of 14.1 [standard deviation (SD), 1.7] years. Overall, 29 subjects were allocated to the Accel-group, 25 to the Accel-sham and 27 to the Fixed-only group. Progress of subjects through the trial is shown in the CONSORT diagram in Fig. 2. A total of 80/81 (99%) and 77/81 (95%) of subjects returned completed questionnaires at T1 and T2, respectively. Missingness was not substantial and therefore classified as missing at random, as it was not dependent on any covariate: of those that were recruited, only 1 was lost at T1 (1 Fixed-only group) and 4 at T2 (1 Accel-group; 1 Sham-group and 2 Fixed-only group).

### Baseline data

Table 1 shows baseline demographics and clinical characteristics of the randomized groups. All three randomized groups were comparable for age, sex, recruitment site and irregularity-index. For the total sample, mean irregularity-index at T0 was 8.5 mm [SD 3.8; 95% CI 7.6 to 9.3] whilst at T2 it was 2.7 mm [SD 2.8; 95% CI 2.2 to 3.4].

### Primary outcome and subgroup analysis

Mean maximum pain intensity across all time-points for the total sample was 72.96 mm [SD 21.59; 95% CI 68.19 to 77.74 mm] with no significant differences among groups (Table 2; P = 0.282). Multivariable regression analysis indicated that even after accounting for all confounders, there were no significant differences between experimental groups (Table 3). However, subject age was marginally significant, with younger subjects reporting higher maximum pain (P = 0.047).

Subgroup analysis according to the use of oral analgesia indicated that subjects taking analgesics reported slightly higher maximum pain intensity compared to those who did not (75.24 mm compared to 68.15 mm, respectively), although this was not statistically significant (Table 2; P = 0.170). The effect of intervention among groups was independent of whether oral analgesia was taken or not (P = 0.883).

### Secondary outcomes

Mean alignment rate, mean pain intensity at each time-point and reported use of oral analgesics amongst the three groups is shown in Supplementary Table S1. The mean overall alignment rate between T1-T2 was 0.10 mm/day (SD 0.05; 95% CI 0.09 to 0.12 mm/day), whilst mean pain intensity varied considerably at the individual time-points after T1 and T2. Overall, a total of 55 (69%) patients at T1 and 26 (34%) patients at T2 reported taking any kind of oral analgesia. For all secondary outcomes studied, no statistically significant difference could be found among the three experimental groups (P > 0.003 due to Bonferroni correction).

**Figure 3:**
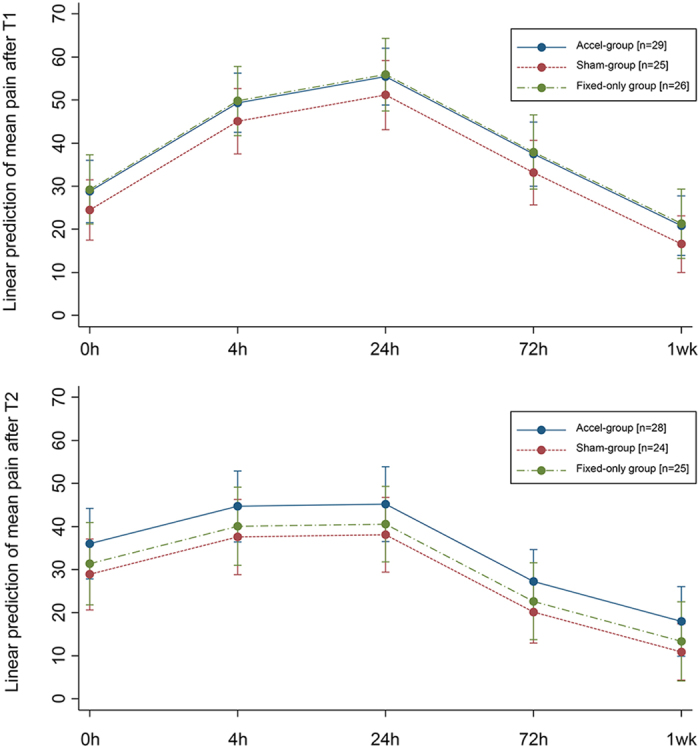
Mean pain intensity at each time-point after T1 and T2 according to intervention (predictive margins of time with 95% CIs).

Multivariable linear regression for mean pain intensity at each time-point (Supplementary Table S2) indicated that the only significant predictor of pain intensity was time, although the amount of irregularity at T2 was marginally significant (P = 0.048). Both after T1 and T2, a statistically and clinically significant increase in pain was seen at 4 and 24 hours following archwire placement, which declined at 72 hours and became insignificant at 1 week (Fig. 3). When taking into account possible confounders through multivariable logistic or linear regression, the use of a functional AcceleDent device or AcceleDent-sham device had no significant effect on the reported use of oral analgesia (Supplementary Table S3) or the number of oral analgesics taken after T1 or T2 (Supplementary Table S4). A marginally significant effect of subject age on number of paracetamol (acetaminophen) taken was seen at T1 (P = 0.043).

No harms were recorded for any subjects within the trial, except pain after insertion of the archwire at T1 and T2.

## Discussion

This randomized controlled trial found no evidence of a significant difference in orthodontic pain during initial tooth alignment between subjects treated with fixed appliances supplemented with the daily application of vibrational force and those treated with fixed appliances alone. Tooth movement and alignment was able to proceed normally in subjects using functional and non-functional vibratory devices with no significant differences between randomized groups. The use of vibrational force had no significant influence on the reported use of analgesics. The only significant predictor of pain intensity was time, with a clinically significant increase in pain being seen at 4 and 24 hours following the placement of alignment archwires at both time-points, which declined at 72 hours and became insignificant at 1 week. Marginally significant effects were identified between maximum reported pain and subject age, mean pain and irregularity at T2, and between subject age and the number of paracetamol taken at T2. However, these were all secondary factors that only just reached statistical significance and were therefore unlikely to be of clinical significance.

This investigation has a number of strengths, which include the multicenter design and strict randomization with concealed allocation, a low drop-out rate amongst recruited subjects and a high percentage return of pain questionnaires. All three randomized groups were comparable for age, sex, recruitment site and irregularity-index. In addition, orthodontic pain experience was measured longitudinally at two important time-points during the alignment phase of routine fixed appliance orthodontic treatment. Although the *a priori* sample size calculation was not based upon the outcome of orthodontic pain^42^, *post hoc* analysis indicated a power of >80% to detect a clinically meaningful difference in pain. Due to the broad inclusion criteria used and the balanced characteristics of the three groups, the results of this trial are applicable to most subjects under 20 years of age undergoing routine orthodontic treatment with fixed appliances for the correction of mandibular crowding. This is the first randomized clinical trial to investigate orthodontic pain in association with use of the market-leading supplemental vibrational device and provides a high-level of evidence to inform clinical practice.

A potential limitation of this study is the absence of definitive compliance data relating to use of the active and sham devices. Both were provided directly by the manufacturer and were fitted with electronic timers designed to measure usage. Unfortunately, these timers proved to be unreliable and obtaining a definitive dataset to inform the analysis was not possible. Subjects were carefully monitored for compliance during the trial, being asked to bring their appliance with them for use prior to each appointment, demonstrate continued familiarity with operation and allow inspection for evidence of use. This was a pragmatic study designed to investigate supplementary vibrational force during routine everyday orthodontic practice as part of the overall management of subjects with malocclusion. The first data-set was obtained during the week immediately following appliance placement and it would be expected that for a simple removable device, compliance levels would be high. A lack of true blinding may also be regarded as a limitation, but the blinding of operators and subjects was not practical. Subjects were blinded to the allocation of functional or sham devices, but the lack of vibration associated with the sham appliance meant that for some, the specific allocation became apparent. However, the randomization of subjects to treatment with a sham device was justified, providing a placebo within the trial, which did not adversely affect drop out rates during the period of investigation.

The use of supplemental vibrational force has been advocated as a simple non-invasive method of improving the efficiency of orthodontic treatment with fixed appliances. In particular, as a method of increasing rates of tooth movement during alignment, leveling and translation^45^,^46^,^47^; however, this evidence is conflicting^38^,^42^. To date, the pain-relieving properties of vibrational therapy during tooth alignment with fixed appliances has only been evaluated in relation to the Tooth Massuese device, and has been found to make no significant difference on the basis of VAS scores^38^. Here, we have focused specifically on pain during the first two phases of alignment, in the first week following appliance placement and following the first adjustment, finding no significant differences between groups. There were no differences between groups in relation to maximum pain at any time-point or the patterns of mean pain during the week following T1 and T2. In general, mean pain peaked within the first few hours after the appointment and then declined to baseline levels at 7 days. These findings are consistent with previous studies of orthodontic pain during the alignment phase of treatment^4^,^7^,^8^,^9^,^17^.

There is evidence that non-prescription oral analgesia can be effective in the management of pain following the activation of fixed orthodontic appliances, particularly nonsteroidal anti-inflammatory drugs at 6 and 24 hours following initial archwire placement^15^. In this investigation, around two-thirds of subjects at T1 and one third at T2 reported the consumption of oral analgesia. However, the use of a functional or sham AcceleDent device had no significant effect on this; or indeed, the number of analgesics taken at either time-point. Therefore, vibrational force does not reduce the levels of pain reported or influence the consumption of oral analgesia.

This study has indicated that supplemental vibrational force provides no added value during initial tooth alignment with fixed appliances in terms of reducing the pain associated with this process. Given that this device is unlikely to be reimbursed in most countries, orthodontists should carefully consider their advice to orthodontic patients considering the use of AcceleDent as a method of pain relief. Current evidence would suggest that in appropriate patients, conventional non-prescription analgesia should be recommended if pain relief is required, with the caveat that pain is likely to reduce after the first 24 hours following archwire ligation and that in general, pain will be insignificant after a week.

This prospective randomized clinical trial has found no evidence that supplemental vibrational force with an AcceleDent removable device can (1) reduce pain; or (2) the consumption of analgesics during the alignment phase of fixed appliance orthodontic treatment.

## Additional Information

**How to cite this article**: Woodhouse, N. R. *et al*. Supplemental vibrational force does not reduce pain experience during initial alignment with fixed orthodontic appliances: a multicenter randomized clinical trial. *Sci. Rep*. **5**, 17224; doi: 10.1038/srep17224 (2015).

## Supplementary Material

Supplementary Information

## Figures and Tables

**Figure 1:**
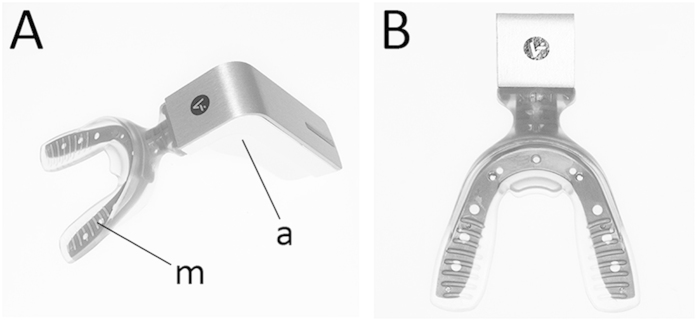
(**A**) The AcceleDent device is a small, lightweight and portable vibrational appliance that consists of an activator unit (a) and a vibrating mouthpiece (m). (**B**) The patient gently bites down on the occlusal surfaces of the vibrating mouthpiece for 20 minutes per day.

**Table 1:** Baseline demographics and characteristics of randomized groups.

Characteristic	Accel-group	Accel-sham	Fixed-only
Age (years)—mean [SD]	13.9 [1.6]	13.8 (1.7)	14.4 [1.9]
Male/female	15/14	13/12	12/15
Trial site, n			
Guy’s	5	5	8
Brighton	11	11	11
Ashford	13	9	8
Irregularity (mm)—mean [95% CI]			
Baseline (T1)	8.3 [6.7–9.9]	8.1 [6.8–9.5]	8.9 [7.4–10.5]
Initial alignment (T2)	2.8 [1.8–3.8]	2.2 [1.4–3.0]	3.3 [1.9–4.7]

SD, standard deviation; CI, confidence interval.

**Table 2:** Reported maximum pain from each patient across all time-points and subgroup analysis according to use of oral analgesia.

Subgroup	Total		P value	Accel-group		Accel-sham		Fixed-only		P value
	n	Mean (SD)		n	Mean (SD)	n	Mean (SD)	n	Mean (SD)	
Overall	81	72.96 (21.59)		29	76.28 (18.86)	25	67.32 (23.81)	27	74.63 (21.95)	0.282*
Oral analgesia: NO	26	68.15 (24.15)	0.170†	8	71.63 (27.42)	10	61.80 (18.33)	8	72.63 (28.38)	0.883‡
Oral analgesia: YES	55	75.24 (20.10)		21	78.05 (14.93)	15	71.00 (26.82)	19	75.47 (19.49)	

SD, standard deviation.

^*^P value for differences among experimental groups from one-way ANOVA.

^†^P value for differences between subgroups NO/YES oral analgesia from independent t-test.

^‡^P value for interaction between use of pain medication and the three experimental groups from two-way ANOVA.

**Table 3:** Multivariable regression for the primary outcome (maximum pain for each patient across all time-points).

	Overall (n = 80)		P value
	Factor	Coefficient (95% CI)	
	Gender	−3.69 (−13.05,5.68)	0.441
	Age	−2.40 (−4.76,−0.03)	0.047
	Irregularity	−0.02 (−1.58,1.54)	0.977
	Oral analgesia use	4.14 (−5.74,14.02)	0.412
	Alignment rate	91.53 (−4.37,187.42)	0.061
Experimental groups	Accel-group	0.14 (−10.06,10.35)	0.978
	Accel-sham	−9.42 (−21.34,2.50)	0.121
	Fixed-only	*Reference*	

Interaction terms: oral analgesia with group: P = 0.715; alignment with group: P = 0.189; irregularity with group: P = 0.123.

CI, confidence interval.
