# Supplementary material for: Supplemental vibrational force does not reduce pain experience during initial alignment with fixed orthodontic appliances: a multicenter randomized clinical trial
Source: Sci Rep. 2015 Nov 27;5:17224. doi: 10.1038/srep17224 (PMC4661602; doi:10.1038/srep17224)
Supplement: Supplementary Information [file srep17224-s1.doc]

**Supplemental vibrational force does not reduce pain experience during initial alignment with fixed orthodontic appliances: a multicenter randomized clinical trial**

Neil R. Woodhouse, Andrew T. DiBiase, Spyridon N. Papageorgiou, Nicola Johnson, Carmel Slipper, James Grant, Maryam Alsaleh, Martyn T. Cobourne

**Supplementary Tables S1-3**

**Supplementary Table S1** Alignment rate, mean pain and analgesia usage at each time-point

|  |  | **Total** | |  | **Accel-group** | | **Accel-sham** | | **Fixed-only** | |  |
| --- | --- | --- | --- | --- | --- | --- | --- | --- | --- | --- | --- |
|  | **Time** | **n** | **Mean (SD)** |  | **n** | **Mean (SD)** | **n** | **Mean (SD)** | **n** | **Mean (SD)** | **P value*** |
| **Alignment rate** | T1 to T2 | 80 | 0.10 (0.05) |  | 29 | 0.10 (0.05) | 25 | 0.11 (0.06) | 26 | 0.10 (0.05) | 0.655 |
| **Mean pain** |  |  |  |  |  |  |  |  |  |  |  |
| Visit 1 | 0 hours | 80 | 27.59 (24.44) |  | 29 | 25.97 (22.80) | 25 | 28.76 (24.68) | 26 | 28.27 (26.74) | 0.905 |
|  | 4 hours | 80 | 48.15 (25.50) |  | 29 | 46.34 (24.65) | 25 | 48.56 (25.46) | 26 | 49.77 (27.29) | 0.882 |
|  | 24 hours | 80 | 54.26 (27.04) |  | 29 | 59.10 (22.39) | 25 | 45.12 (28.89) | 26 | 57.65 (28.71) | 0.122 |
|  | 72 hours | 80 | 36.26 (27.59) |  | 29 | 40.14 (29.50) | 25 | 28.88 (24.54) | 26 | 39.04 (27.80) | 0.272 |
|  | 1 week | 80 | 19.65 (22.87) |  | 29 | 22.03 (23.24) | 25 | 15.68 (19.14) | 26 | 20.81 (25.93) | 0.573 |
|  |  |  |  |  |  |  |  |  |  |  |  |
| Visit 2 | 0 hours | 77 | 32.14 (28.31) |  | 28 | 31.68 (28.61) | 24 | 30.88 (25.35) | 25 | 33.88 (31.57) | 0.930 |
|  | 4 hours | 77 | 40.88 (28.98) |  | 28 | 47.21 (30.52) | 24 | 38.00 (28.31) | 25 | 36.56 (27.73) | 0.349 |
|  | 24 hours | 77 | 41.44 (29.47) |  | 28 | 53.18 (31.18) | 24 | 34.96 (28.13) | 25 | 34.52 (25.39) | 0.028 |
|  | 72 hours | 77 | 23.60 (23.34) |  | 28 | 27.43 (23.44) | 24 | 21.88 (20.90) | 25 | 20.96 (25.72) | 0.554 |
|  | 1 week | 77 | 14.36 (24.27) |  | 28 | 18.43 (28.88) | 24 | 9.17 (9.93) | 25 | 14.80 (28.03) | 0.393 |
| **Analgesia usage** |  |  |  |  |  |  |  |  |  |  |  |
| Visit1 |  | 80 | 55/80 (69%) |  |  | 21/29 (72%) |  | 15/25 (60%) |  | 19/26 (73%) | 0.533 |
| Visit 2 |  | 77 | 26/77 (34%) |  |  | 9/28 (32%) |  | 9/24 (38%) |  | 8/25 (32%) | 0.901 |
| **Ibuprofen taken** |  |  |  |  |  |  |  |  |  |  |  |
| Visit1 |  | 80 | 0.53 (1.18) |  |  | 0.45 (0.74) |  | 0.16 (0.47) |  | 0.96 (1.80) | 0.046 |
| Visit 2 |  | 77 | 0.21 (0.80) |  |  | 0.04 (0.19) |  | 0.50 (1.32) |  | 0.12 (0.44) | 0.090 |
| **Paracetamol taken** |  |  |  |  |  |  |  |  |  |  |  |
| Visit1 |  | 80 | 1.55 (2.43) |  |  | 1.62 (3.09) |  | 1.04 (1.27) |  | 1.96 (2.44) | 0.397 |
| Visit 2 |  | 77 | 0.52 (1.02) |  |  | 0.64 (1.16) |  | 0.54 (1.02) |  | 0.36 (0.86) | 0.604 |

SD,standard deviation.

*P value for differences among the three experimental groups from one-way ANOVA (except for analgesia use, where chi-square test was employed). Due to the application of Bonferroni correction for the existence of 17 tests, P values < 0.003 are regarded as significant

**Supplementary Table S2** Multivariable regression of the secondary outcome mean pain at T1 and T2

|  | **T1 (n=80)** | |  | **T2 (n=77)** | |
| --- | --- | --- | --- | --- | --- |
|  | **Coefficient (95% CI)** | **P value** |  | **Coefficient (95% CI)** | **P value** |
| Gender | -3.24 (-10.85,4.37) | 0.404 |  | -7.02 (-15.81,1.76) | 0.117 |
| Age | -0.06 (-2.31,2.19) | 0.959 |  | -1.34 (-3.89,1.20) | 0.300 |
| Irregularity | -0.06 (-1.14,1.02) | 0.913 |  | 1.05 (0.01,2.10) | 0.048 |
| Painkiller use | 3.06 (-5.85,11.98) | 0.501 |  | 1.12 (-6.45,8.69) | 0.772 |
|  |  |  |  |  |  |
| Accel-group | -0.18 (-9.46,-9.10) | 0.970 |  | 5.00 (-6.13,16.14) | 0.378 |
| Accel-sham | -4.11 (-13.63,5.42) | 0.398 |  | -1.89 (-12.61,8.83) | 0.730 |
| Fixed-only | *Reference* |  |  | *Reference* |  |
|  |  |  |  |  |  |
| 0 hours | *Reference* |  |  | *Reference* |  |
| 4 hours | 20.56 (14.04,27.08) | <0.001 |  | 8.68 (2.50,14.85) | 0.006 |
| 24 hours | 26.68 (18.79,34.56) | <0.001 |  | 9.17 (1.99,16.35) | 0.012 |
| 72 hours | 8.68 (1.19,16.16) | 0.023 |  | -8.74, -14.84,-2.63) | 0.005 |
| 1 week | -7.94 (-14.00,-1.88) | 0.010 |  | -18.04 (-24.62,11.46) | <0.001 |

Interaction term of time with group: after 1rst visit: P=0.566; after 2^nd^ visit: P=0.549.

CI, confidence interval.

**Supplementary Table S3** Multivariable logistic regression of the secondary outcome use of analgesia

|  |  | **Odds Ratio (95% CI)** | **P value** |
| --- | --- | --- | --- |
| T1 | Gender | 0.70 (0.26,1.91) | 0.485 |
|  | Age | 0.81 (0.61,1.09) | 0.164 |
|  | Irregularity | 1.02 (0.90,1.15) | 0.810 |
|  |  |  |  |
|  | Accel-group | 0.92 (0.27,3.12) | 0.891 |
|  | Accel-sham | 0.51 (0.15,1.73) | 0.280 |
|  | Fixed-only | *Reference* |  |
|  |  |  |  |
| T2 | Gender | 1.04 (0.40,2.68) | 0.942 |
|  | Age | 0.91 (0.72,1.29) | 0.800 |
|  | Irregularity | 0.98 (0.87,1.12) | 0.874 |
|  |  |  |  |
|  | Accel-group | 0.99 (0.31,3.13) | 0.987 |
|  | Accel-sham | 1.25 (0.38,4.12) | 0.718 |
|  | Fixed-only | Reference |  |

CI, confidence interval.

**Supplementary Table S4** Multivariable regression of the secondary outcome number of pain analgesics taken

|  |  | **Ibuprofen** |  |  | **Paracetamol** |  |
| --- | --- | --- | --- | --- | --- | --- |
|  |  | **Coefficient (95% CI)** | **P value** |  | **Coefficient (95% CI)** | **P value** |
| T1 | Gender | -0.08 (-0.53,0.37) | 0.732 |  | 0.08 (-0.93,1.09) | 0.879 |
|  | Age | -0.05 (-0.16,0.07) | 0.418 |  | -0.20 (-0.39,-0.01) | 0.043 |
|  | Irregularity | -0.04 (-0.08,0.01) | 0.140 |  | 0.03 (-0.08,0.13) | 0.598 |
|  |  |  |  |  |  |  |
|  | Accel-group | -0.55 (-1.28,0.17) | 0.136 |  | -0.43 (-1.83,0.97) | 0.550 |
|  | Accel-sham | -0.85 (-1.55,-0.16) | 0.016 |  | -1.02 (-2.10,0.06) | 0.064 |
|  | Fixed-only | *Reference* |  |  | *Reference* |  |
|  |  |  |  |  |  |  |
| T2 | Gender | 0.19 (-0.17,0.55) | 0.290 |  | -0.06 (-0.51,0.38) | 0.784 |
|  | Age | -0.02 (-0.09,0.05) | 0.573 |  | 0.01 (-0.09,0.11) | 0.887 |
|  | Irregularity | 0.07 (-0.03,0.16) | 0.187 |  | -0.01 (-0.08,0.06) | 0.769 |
|  |  |  |  |  |  |  |
|  | Accel-group | -0.10 (-0.33,0.14) | 0.417 |  | 0.29 (-0.24,0.82) | 0.287 |
|  | Accel-sham | 0.40 (-0.14,0.94) | 0.148 |  | 0.18 (-0.36,0.72) | 0.507 |
|  | Fixed-only | *Reference* |  |  | *Reference* |  |

CI, confidence interval.
